# Glottic Spasm

**Published:** 1891-04-11

**Authors:** 


### SURGERY.

### GLOTTIC SPASM.

The operation of stretching or of division of the superior laryngeal nerve for spasmodic closure of the glottis has been advocated by Dr. Creveling. Of course such a procedure is only adopted when the cause of glottic spasm is a more or less irremediable and peristent condition. Dr. Creveling has reported two cases in which this suggestion was practically applied in practice. The first was one of division of the superior laryngeal nerve, which became necessary from the reflex disturbance in respiration and glottic spasm, resulting from a tumour involving the nerve. In the second case the nerve was simply cut down to and exposed and stretched. In each of these cases the result was excellent, and the glottic spasm prevented, and this warrants the assumption that in certain cases the superior laryngeal nerve is an important factor in spasmodic diseases of the larynx, and that irritation of its terminal fibres, as well as irritation of the trunk of the nerve, may give rise to spasm of the glottis; that a disturbance of this nerve may be reflected to other parts supplied by the pneumogastric nerve, and *vice versa;* that division of the superior laryngeal nerve may not be followed by serious results in the larynx; that proper tension applied to the trunk of the nerve may modify its influence or action on the parts to which it is distributed. Under certain circumstances it may be advisable to stretch or divide the nerve, and the operation should not be performed except to relieve some extreme condition not amenable to other treatment.

